# Lumbar spinal stenosis exacerbated by spinal epidural lipomatosis: a case report

**DOI:** 10.3389/fsurg.2026.1770604

**Published:** 2026-02-10

**Authors:** Yide Fang, Zhenghang Bian, Zhengyi Tong, Jinhai Xu, Wen Mo

**Affiliations:** Department of Orthopedics, Longhua Hospital, Affiliated to Shanghai University of Traditional Chinese Medicine, Shanghai, China

**Keywords:** case report, lumbar spinal stenosis, recovery kinetics, selective nerve root block, spinal epidural lipomatosis

## Abstract

**Objective:**

To characterize the presentation, diagnostic strategy, and postoperative recovery patterns in a patient with lumbar spinal stenosis (LSS) complicated by spinal epidural lipomatosis (SEL), and to evaluate the role of selective nerve root block (SNRB) in guiding staged minimally invasive decompression.

**Methods:**

We analyzed the clinical course of a 65-year-old man presenting with neurogenic claudication and right lower extremity numbness after a course of inhaled glucocorticoids. Preoperative assessment included radiography, computed tomography (CT), and magnetic resonance imaging (MRI), which demonstrated degenerative central canal stenosis at L4/5 and epidural lipomatosis at L5/S1. Because imaging findings were multilevel and symptom dominance was unclear, SNRB localized the symptomatic level and guided staged arthroscopy-assisted uniportal decompression. Clinical outcomes were assessed using the Visual Analog Scale (VAS), Oswestry Disability Index (ODI), SF-36 quality-of-life scores, walking tolerance, and neurological examinations during 4 months of follow-up.

**Results:**

SNRB identified L4/5 as the primary symptomatic level, supporting initial targeted decompression. Following Stage 1 decompression at L4/5, neurogenic claudication and motor weakness improved rapidly, with VAS decreasing from 7 to 4 and ODI improving from 52 to 38. Persistent plantar numbness prompted Stage 2 decompression with excision of excessive epidural fat at L5/S1, after which VAS further decreased to 2 and ankle plantarflexion strength improved relative to baseline. At the final follow-up, ODI improved to 20 and the SF-36 composite score increased from 32.6 to 70.8. Walking tolerance markedly improved, only mild plantar paresthesia persisted. No perioperative complications or postoperative instability were observed.

**Conclusions:**

This case suggests that SEL may trigger or unmask clinical symptoms of degenerative LSS and highlights distinct recovery trajectory between focal degenerative stenosis and diffuse epidural fat–related compression. SNRB-guided staged minimally invasive decompression enabled precise level selection and favorable functional outcomes, underscoring its value in managing multilevel lumbar pathology complicated by SEL.

## 1 Introduction

Lumbar spinal stenosis (LSS) is a prevalent degenerative spinal disorder characterized by narrowing of the spinal canal or lateral recess, resulting in compression of neural elements and symptoms such as low back pain, radiculopathy, weakness, and neurogenic claudication (1). Degenerative changes including disc bulging, facet joint hypertrophy, and ligamentum flavum thickening represent the most common causes (2, 3). In contrast, spinal epidural lipomatosis (SEL) is an uncommon condition defined by excessive accumulation of unencapsulated epidural adipose tissue, most frequently associated with obesity, chronic corticosteroid exposure, or endocrine disorders (4). Although SEL may be asymptomatic, progressive fat deposition can produce clinically significant neural compression, ranging from chronic radiculopathy to acute cauda equina syndrome (5). When SEL coexists with degenerative LSS, its contribution to symptom onset and severity is often underestimated.

The coexistence of LSS and SEL poses diagnostic and therapeutic challenges, as imaging abnormalities are frequently multilevel and symptom severity does not necessarily correlate with degenerative stenosis alone (6). Increasing evidence suggests that SEL may act as a precipitating factor, converting compensated or subclinical degenerative stenosis into clinically overt or acute LSS (7). However, most published reports rely primarily on imaging-based decision-making and describe single-stage decompression, with limited functional localization or analysis of postoperative recovery patterns (8, 9). The present case is unique in providing a rare within-patient comparison of recovery kinetics following decompression of focal degenerative stenosis vs. diffuse epidural fat–related compression, guided by selective nerve root block (SNRB). This observation offers mechanistic insight into symptom precipitation and differential neurological recovery in LSS complicated by SEL.

## 2 Case presentation

A 65-year-old man presented with a 2-month history of progressive low back pain with right lower extremity numbness and neurogenic claudication. The patient could ambulate approximately 500 m before neurogenic claudication required rest. He reported a pulling sensation over the posterolateral right calf, with paresthesia involving the dorsum and plantar surface of the foot. Two months prior to symptom onset, the patient received nebulized budesonide (1 mg in 2 mL) twice daily for 13 days (cumulative dose, 26 mg), in combination with nebulized ambroxol and procaterol for allergic laryngeal edema. He did not receive systemic (oral or intravenous) glucocorticoids. He denied bowel or bladder dysfunction. Prior conservative treatment (acupuncture and mecobalamin) provided limited benefit. His body mass index was 26.8 kg/m². He denied hypertension, diabetes mellitus, coronary artery disease, and other major medical comorbidities. He had no history of lumbar surgery and was a non-smoker. At baseline, the patient had severe pain with a Visual Analog Scale score of 7, marked disability with an Oswestry Disability Index score of 52, reduced quality of life with an SF-36 score of 32.6, and pronounced plantar numbness rated as 6 on a 0–10 scale. Details are provided in Table 1.

**Table 1:** Timeline of clinical outcomes and interventions in the present patient.

Timepoint (date)	Clinical stage/follow-up	VAS	ODI	SF-36 (composite)	Walking distance (m)	Numbness severity (0–10)
2025-07-28	Preoperative	7	52	32.6	500	6
2025-08-01	Stage 1, postoperative day 3	4	38	40.1	1,000	4
2025-10-10	Stage 2, postoperative day 3	2	30	56.3	1,200	3
2025-12-20	Final follow-up	1	20	70.8	2,000	3

VAS, visual analog scale; ODI, Oswestry disability index; SF-36, 36-item short form health survey.

**Figure 1:**
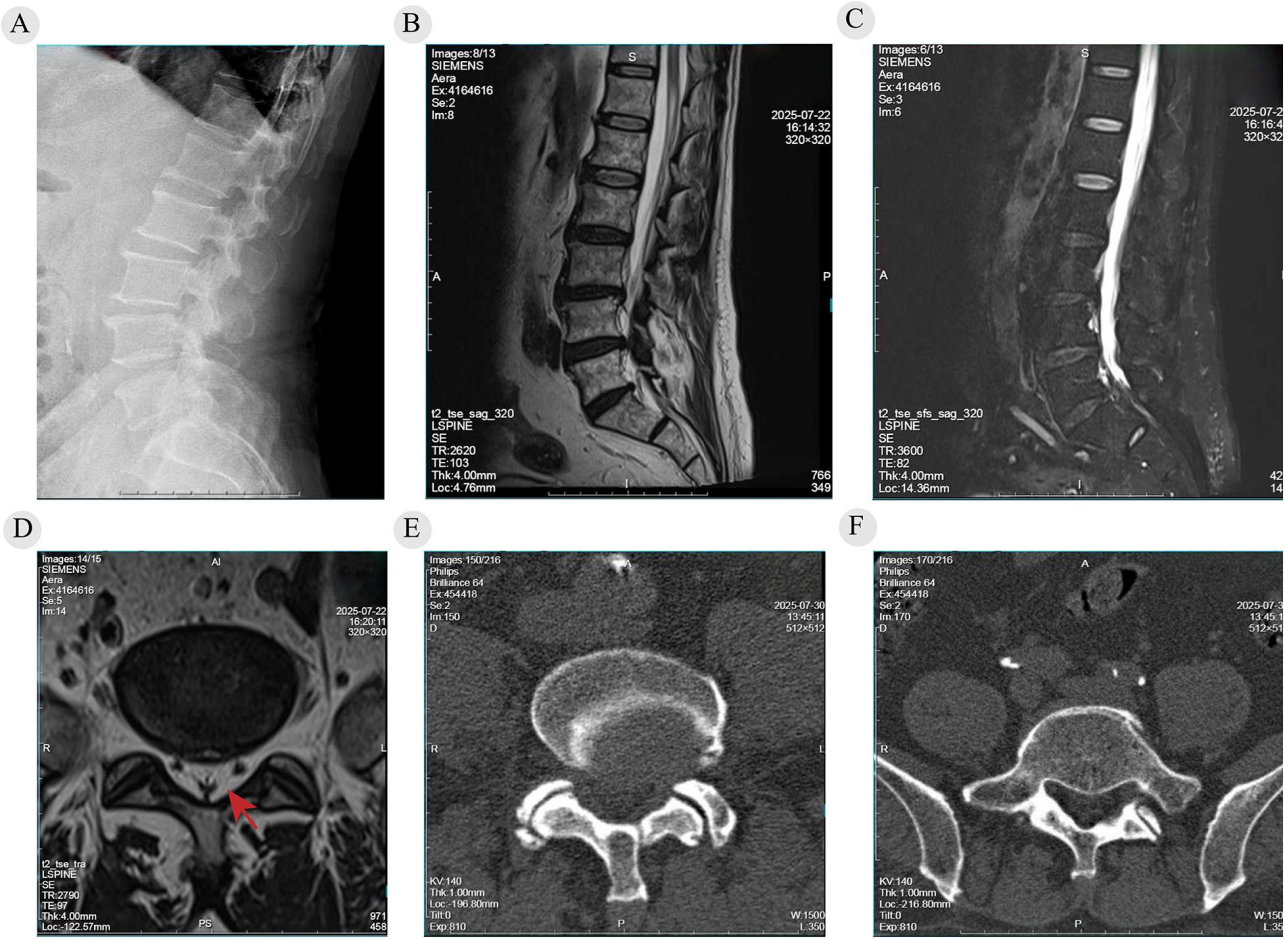
Preoperative imaging findings and key features of LSS complicated by SEL. **(A)** Lateral lumbar radiograph showing degenerative disc disease with reduced intervertebral disc height. **(B)** Sagittal T1-weighted MRI revealing central canal stenosis at L4–L5 and mild disc protrusion at L3/4 and L4/5. **(C)** Sagittal T2-weighted fat-suppressed MRI demonstrating marked posterior epidural lipomatosis from L4 to S1 and ligamentum flavum hypertrophy at L4/5. **(D)** Axial T2-weighted MRI at L5/S1 showing severe circumferential compression of the thecal sac by excessive epidural fat, forming the typical “Y-sign” (red arrow). **(E)** Axial CT at L4/L5 showing degenerative osseous changes without calcification. **(F)** Axial CT at L5/S1 confirming no calcification and outlining the lumbosacral osseous canal morphology. Written consent for publication was obtained.

Physical examination revealed markedly restricted lumbar extension with mild paraspinal tenderness to palpation and percussion at L4/5 and L5/S1. Sensation was decreased over the posterolateral right calf, dorsum, and plantar foot. Motor testing showed Medical Research Council (MRC) grade 4/5 weakness of the right extensor hallucis longus (EHL) and ankle plantarflexion. Other muscle groups were normal. Straight-leg raise and femoral nerve stretch tests were negative bilaterally. Lower extremity tendon reflexes were diminished, and pathological reflexes were negative. Preoperative radiographs and CT demonstrated degenerative changes. MRI showed significant canal stenosis at L4/5 with multilevel disc protrusions (L3/4 and L5/S1) and prominent epidural lipomatosis at L5/S1 compressing the dural sac with a characteristic Y-sign on axial imaging (Figure 1). Because imaging alone could not confidently localize the symptomatic level in multilevel disease, SNRB was used to guide staged decompression. SNRB was performed under fluoroscopic guidance. The L5 nerve root was accessed via a transforaminal route and the S1 root through the sacral foramen. When radiating leg pain was elicited, 0.5 mL of contrast was injected to confirm root trajectory. Subsequently, a mixture of 3 mg dexamethasone and 4 mL of 0.8% lidocaine was administered. Injection induced temporary symptoms similar to the patient's usual pain, indicating accurate targeting. After the block, the patient remained supine for 1 h for observation. Pain relief ≥50% was considered a positive block. The overall symptom profile supported prioritizing L4/5 as the first-stage target.

**Figure 2:**
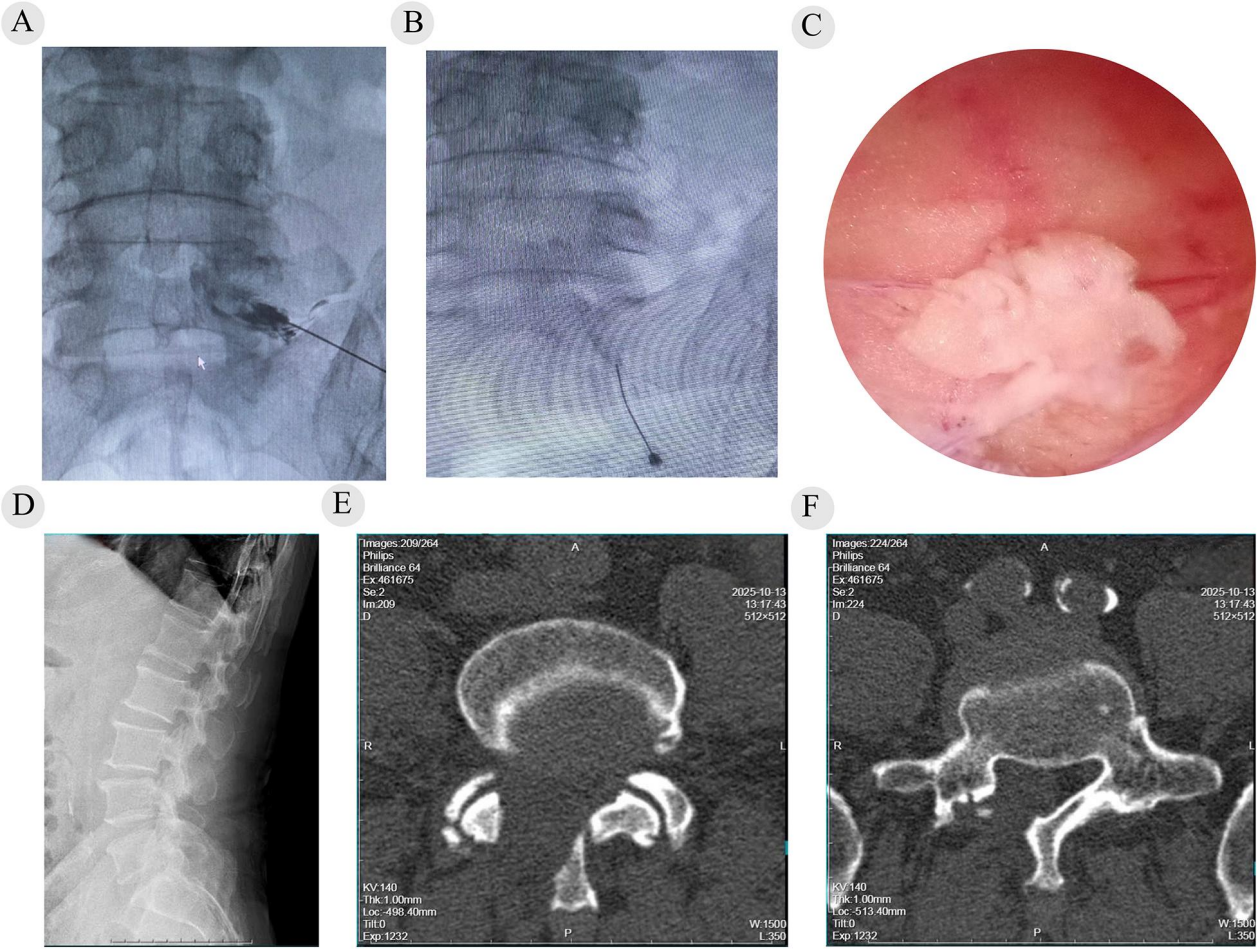
Intraoperative and postoperative findings. **(A)** Anteroposterior fluoroscopic view during SNRB at the L5 foramen, demonstrating contrast opacification outlining the L5 nerve root. **(B)** Fluoroscopic view during SNRB at the S1 sacral foramen, showing contrast spread along the S1 nerve root. **(C)** Intraoperative endoscopic (arthroscopy-assisted) view during Stage II L5/S1 decompression, demonstrating excessive posterior epidural fat. **(D)** Postoperative lateral lumbar radiograph. **(E,F)** Postoperative axial CT images at L4/5 and L5/S1, respectively, demonstrating adequate decompression. Written consent for publication was obtained.

Based on clinical and imaging findings, the patient was diagnosed with lumbar spinal stenosis at L4/5 combined with spinal epidural lipomatosis at L5/S1. A right L5 SNRB performed under fluoroscopic guidance reproduced and partially relieved his typical symptoms, supporting an initial, targeted decompression. Stage 1 surgery consisted of arthroscopy-assisted uniportal decompression at L4/5 with right L5 nerve root release. The procedure was performed through a skin incision approximately 1.5–2.0 cm. No fixed working channel was established. Instead, a limited operative space was created to allow endoscopic manipulation. A ShenDa endoscopic system was used. The operative time for Stage 1 was approximately 100 min, with an estimated blood loss of 20 mL. Postoperatively, claudication and calf traction pain improved, and the pain score decreased to VAS 4. Right EHL strength recovered to grade V, but plantar numbness persisted. Given residual plantar symptoms and persistent L5/S1 compression on imaging, an additional S1 SNRB was performed, after which the patient reported partial improvement in plantar numbness. Stage 2 arthroscopy-assisted uniportal bilateral decompression at L5/S1 was performed, with resection of epidural fat and adequate decompression of the dural sac and nerve roots (Figure 2). Stage 2 decompression required approximately 190 min, with an estimated blood loss of 50 mL. Decompression was confirmed under endoscopic visualization. Specimens obtained during the second procedure were submitted for histopathological examination. Grossly, the ligamentum flavum specimen measured approximately 35 × 15 × 3 mm, and the epidural specimen measured approximately 12 × 8 × 4 mm. Histopathological examination revealed that the ligamentum flavum showed degenerative fibrous tissue with focal calcification, while the epidural specimen consisted of fibrous tissue and mature adipose tissue, supporting the diagnosis of spinal epidural lipomatosis. After Stage 2, ankle plantarflexion improved to grade V, plantar numbness was markedly alleviated, and pain decreased to a VAS score of 2. At the 2-month follow-up, low back pain and claudication were nearly resolved, with only mild residual plantar paresthesia not affecting daily activities. No perioperative complications were observed. The patient tolerated both procedures well and adhered to postoperative rehabilitation. The patient reported that preoperatively the main limitations were walking intolerance due to neurogenic claudication and persistent right plantar numbness, which interfered with daily activities. After staged surgery, he noted substantial improvement in walking capacity and pain. Following the second procedure, plantar numbness improved substantially. Leg strength and walking capacity improved noticeably, and residual symptoms no longer affected daily life. The longitudinal changes in VAS, ODI, SF-36, walking distance, and numbness severity are summarized in Table 1, showing progressive functional recovery following staged decompression.

## 3 Discussion

SEL is increasingly recognized as a clinically relevant modifier of degenerative LSS rather than an incidental imaging finding. Although SEL remains relatively uncommon in the general population, its prevalence is substantially higher among patients undergoing surgery for degenerative lumbar disease (5, 10). Epidemiological studies have identified obesity, male sex, metabolic disorders, and exogenous corticosteroid exposure as the principal risk factors (11). Notably, the anatomical distribution of SEL varies by etiology: steroid-related SEL more frequently involves the thoracic spine, whereas obesity-related and idiopathic SEL predominantly affect the lumbosacral region (12). Clinically, SEL may produce symptoms ranging from chronic radiculopathy and neurogenic claudication to acute cauda equina syndrome (13). However, when SEL coexists with multilevel degenerative changes, its contribution to symptom onset is often underestimated, and clinical deterioration may be incorrectly attributed to progression of degenerative stenosis alone. In this context, increasing evidence supports the concept that SEL acts as a “compression threshold amplifier,” converting compensated or subclinical stenosis into clinically overt or even acute LSS. In the present case, a short course of inhaled glucocorticoids, together with overweight status and an adverse metabolic milieu, likely accelerated epidural fat expansion at L5/S1, precipitating symptom onset in a spine already compromised by degenerative narrowing at L4/5. Although the present exposure was a short course of nebulized budesonide and is not equivalent to systemic steroid therapy, prior reports have suggested that even brief steroid exposure may be temporally associated with rapid clinical deterioration in susceptible patients (14, 15). Therefore, in our case, glucocorticoid exposure is discussed as a potential unmasking factor rather than evidence of causality.

Most previously reported cases of SEL involving the L5–S1 level were treated with single-stage laminectomy (16, 17). In contrast to these reports, the present case has two distinct contributions. First, SNRB was used to accurately localize the symptomatic level and guide surgical decision-making. Second, staged minimally invasive decompression was performed at two different levels within the same patient, enabling a within-subject comparison of postoperative recovery kinetics after relief of focal vs. diffuse neural compression. Accurate identification of the responsible level is particularly challenging in patients with combined LSS and SEL, as imaging findings are frequently multilevel and symptom severity does not correlate linearly with canal diameter or fat volume alone (8). SNRB therefore plays an important adjunctive role in functional localization. SNRB was originally developed as a diagnostic tool to resolve discrepancies between clinical symptoms and imaging findings and has since been widely used to guide surgical decision-making in multilevel degenerative disease (18). Systematic reviews have demonstrated that SNRB can improve diagnostic confidence, reduce unnecessary decompression levels, and help predict surgical outcomes when appropriately applied (19). In the present case, right L5 SNRB reproduced and partially relieved the patient's typical symptoms, confirming L5 as the dominant symptomatic root despite concomitant S1-related sensory complaints. This functional confirmation justified an initial targeted decompression at L4/5 rather than empirical multilevel surgery. Importantly, SNRB also served as a dynamic diagnostic tool, revealing persistent S1 involvement after the first operation and supporting the decision to proceed with staged L5/S1 decompression. Compared with most published SEL case reports, which rely primarily on imaging severity to determine surgical extent, this approach highlights the value of integrating functional testing into surgical planning. Such a strategy may be particularly relevant in minimally invasive or endoscopic procedures, where precise level selection is critical to achieving symptom relief while minimizing surgical morbidity (20).

After L4/5 decompression, the patient experienced rapid improvement in neurogenic claudication and motor weakness, which is consistent with contemporary pathophysiologic frameworks of degenerative LSS emphasizing activity-dependent mechanical narrowing with superimposed vascular compromise that can produce predominantly functional conduction impairment when structural injury is limited (21). Following decompression of focal mechanical stenosis, pain severity and walking capacity typically show rapid improvement. In contrast, SEL reflects excessive deposition of unencapsulated epidural fat that narrows the canal and compresses neural elements more diffusely, often in a circumferential pattern. Recent literature further highlights its associations with obesity and its associations with obesity and metabolic syndrome, as well as with steroid exposure, supporting SEL as a distinct compressive phenotype rather than a simple variant of degenerative stenosis (5). Importantly, emerging advanced MRI evidence in compressive lumbar disorders supports a microstructural substrate for delayed or incomplete sensory recovery: diffusion-based metrics of the cauda equina have been shown to correlate with compression-related dysfunction and postoperative trajectories, providing contemporary imaging support for demyelination risk under sustained compression and for differential recovery dynamics after decompression (22). In recent surgical series of SEL, decompression significantly improves pain-free walking distance and patient-reported symptoms, and continued recovery between early postoperative assessment and later follow-up has been documented, but recovery of neurological deficits such as numbness and sensory impairment is substantially slower than that of pain, and residual sensory abnormalities have been reported in some patients even after follow-up periods of up to three years (8, 23, 24). Consistent with these observations, emerging minimally invasive evidence from endoscopic case reports and small case series-including percutaneous full-endoscopic uniportal decompression and unilateral biportal endoscopic decompression-also documents rapid pain and functional improvement, while underscoring that sensory recovery may remain protracted and that long-term outcomes still require further follow-up (25). Previous reports have demonstrated that intraoperative measurements in patients with lumbar epidural lipomatosis show significantly elevated epidural pressure (13). Such circumferential compression is prone to induce more severe venous congestion and tissue edema, thereby aggravating neural ischemia, which in turn can lead to axonal injury and myelin degeneration. Prognostic studies have further shown that the severity of preoperative numbness, the duration of neural compression, patient age, and the presence of metabolic comorbidities are closely associated with the rate of myelin regeneration and with persistent postoperative numbness and sensory disturbance (26, 27). Sensory fibers, particularly those transmitting deep plantar sensation, are highly sensitive to ischemic stress and tend to recover more slowly than motor fibers. Mechanistically, sensory axons exhibit lower tolerance to ischemia and sustained compressive stress, and their ion channel distribution and energy metabolic characteristics render them more susceptible to depolarization and irreversible injury. This vulnerability may explain why recovery of numbness and sensory abnormalities is markedly delayed compared with improvements in pain and motor function in lumbar epidural lipomatosis and other chronic compressive disorders (28). Taken together, we interpret the divergent postoperative course in our patient as hypothesis-generating. Focal degenerative compression at L4/5 may have produced largely reversible functional impairment, whereas circumferential epidural fat–related compression at L5/S1 may have imposed more sustained ischemic stress and microstructural vulnerability, contributing to slower improvement in plantar numbness despite adequate decompression.

However, several limitations should be acknowledged. As a single-case study, the generalizability of the proposed precipitating role of SEL and the observed recovery kinetics is limited, and larger multicenter studies with longer follow-up are needed for confirmation. Moreover, postoperative imaging was limited to CT. While CT can confirm the adequacy of bony decompression, the absence of postoperative MRI limited our ability to objectively evaluate postoperative changes in epidural fat and neural restoration, and to perform robust radiographic–clinical correlation. In addition, postoperative outcomes were jointly assessed by the operating surgeon and a resident physician who was not involved in the surgery. Although complete blinding was not possible, the use of standardized, quantitative measures reduced potential observer bias.

## 4 Conclusion

This case illustrates that SEL can precipitate the clinical onset of LSS by increasing neural compression in a spine already compromised by degenerative changes. Functional localization with SNRB enabled precise, staged decompression in the presence of multilevel pathology. The distinct recovery patterns observed-rapid improvement after decompression of degenerative stenosis and delayed sensory recovery following epidural fat excision-reflect fundamental differences in compression mechanisms. Recognizing these differences is essential for surgical planning and for setting realistic expectations regarding postoperative neurological recovery in patients with LSS complicated by epidural lipomatosis.

## Data Availability

The original contributions presented in the study are included in the article/Supplementary Material, further inquiries can be directed to the corresponding author.
